# The opportunity and imperative for point-of-care diagnostics in systemic lupus erythematosus

**DOI:** 10.1186/ar4647

**Published:** 2014-09-18

**Authors:** Marvin J Fritzler

**Affiliations:** 1 University of Calgary, AB, Canada

## Background

The development of point-of-care diagnostics (POCD) is prompted by the global burden of diseases attended by high morbidity and mortality when the diagnosis is delayed. POCD are designed to bring diagnostic tests immediately accessible to the site of patient care. In general terms, these devices are simple to use, economical, have high specificity and have a short turnaround time, enabling rapid clinical decisions. Contemporary POCD devices target blood glucose, coagulation, cardiac markers, infectious diseases, pregnancy and cholesterol testing. Systemic lupus erythematosus (SLE) is a suitable target for POCD because it can present with a variety of rapid-onset and life-threatening features, and is characterized by a number of well-defined disease-specific biomarkers. Clinical scenarios where POCD would benefit the management of SLE include CNS lupus (psychosis, encephalitis, stroke, seizures), transverse myelitis, acute renal failure, pulmonary hemorrhage and pneumonitis, cytopenias (thrombocytopenia, anemia, neutropenia, leukopenia), catastrophic antiphospholipid syndrome, cardiac tamponade and infarction, fetal heart block, mesenteric vasculitis and pancreatitis.

## Methods

In collaboration with Dr Fooke Laboratorien GmbH (Neuss, Germany) we are developing and beta testing a lateral flow POCD that detects anti-dsDNA within 20 minutes after application of 10 μl serum. Twenty preselected SLE sera and age and gender-matched controls were tested. Interpretation of the test results were performed by direct visual observation or by densitometry using a portable lateral flow assay reader. Semi-quantitative results were expressed as relative units.

## Results

When compared with results of the conventional *Crithidia lucilliae *assay, the POCD had a sensitivity of 90% and a specificity of 100%. Further testing to establish sensitivity, specificity and receiver operator characteristic in unselected SLE and control sera are underway.

## Conclusions

Pilot data using this lateral flow POCD were favorable and had similar characteristics as those recently published by N Offermann in the *Journal of Immunological Methods*. The features of future POCD are based on advanced diagnostic platforms such as lateral flow, novel nanomaterials and microfluidics (lab on a chip), which are miniaturized and portable, have high specificity and design, are multiplexed and high throughput, and are standardized.

